# Efficacy and safety of hepatitis C direct-acting protease inhibitors (DAA-PI ) in real life in HIV-HCV coinfected patients

**DOI:** 10.1186/1471-2334-14-S2-P84

**Published:** 2014-05-23

**Authors:** A Ménard, C Dhiver, I Ravaux, J Moreau, S Mokhtari, P Colson, L Meddeb, P Brouqui, A Stein

**Affiliations:** 1 SMIT, La Conception University Hospital, Marseille, France; 2 SMIT, North University Hospital, Marseille, France; 3 Microbiology federation, Aix Marseille University, URMITE, UM63, CNRS 7278, IRD 198, Inserm 1095, Timone University Hospital, Marseille, France

## Background

HCV Triple therapy opens new perspectives for HCV cure in HIV-HCV patients but data concerning the use of HVC DAA-PI in this patients in a real life setting are scarce. Our objective was to evaluate efficacy and safety of Telaprevir (TLV) and Boceprevir ( BOC) based- therapy in our cohort.

## Materials and methods

We included HIV-HCV patients treated with Peg Interferon RIB (PR) plus TLV/BOC between 2011-2013 in a multicenter retrospective cohort. Demographic, clinical and imunologic characteristics were obtained, as well as adverse events and discontinuations. Treatment efficacy and safety data were collected at week (W) 4, 8, 12, 24 and 48 . Success if HCV-RNA became undetectable (PCRHVC-)72 weeks after the end of treatment (SVR). Failure if treatment was discontinued due to virologic failure (VF), adverse events (AE).

## Results

31 patients were analyzed, 5 Naives and 26 re-treatments (21% relapsers, 53% non responders and 26% intolerance discontinuation). All were Caucasian, 86% were male with a 49 years old’s mean age. Mode of transmission of HIV& HCV was IUDV for 24 patients, MSM for1. Median CD4 counts 692. All were on HAART with undetectable Viral Load in 92%. Background HIV therapy contained in 51% raltegravir (RAL), 20% ritonavir-boosted atazanavir (ATV/r), 12% etravirine (ETV) 9% darunavir (DRV/r),6% lopinavir, efavirenz and rilpivirine. Tenofovir in 72%. Genotype 1a (n=20) or 1b (n=10) or 4 (n=1), METAVIR fibrosis stage was F3F4 in 55%, F2 in 32% and F0F1 in 13% of cases. HVC was treated with PR+ BOC (33%) or TLV (67%). 25 patients reached W72. PCRVHC- was observed in 54% of patients at W4, 67% at W12, 58% at W24 and 58% at W48. SVR was obtained for 58% (50% on PR+BOC, 64% on PR+TLV) and in 51% of cirrhotic patients. Six patients discontinued therapy for VF (n=1) or AE (n=5).

## Conclusion

Despite SVR increased with DAA-PI based therapy, treatment of HCV in HIV remains complex with multiple challenges, including high pill burden, higher rates of adverse events (AEs) and difficult drug-drug-interactions.

